# Genome-Wide and Transcriptome Analysis of Autophagy-Related ATG Gene Family and Their Response to Low-Nitrogen Stress in Sugar Beet

**DOI:** 10.3390/ijms252211932

**Published:** 2024-11-06

**Authors:** Rongli Jia, Ruxin Zhou, Yue Chang, Lei Wei, Liuxi Yi, Binjie Ma, Shude Shi

**Affiliations:** 1 College of Agriculture, Inner Mongolia Agricultural University, Hohhot 010000, China; jiarongli00@163.com (R.J.); zhrx_137@163.com (R.Z.); changyue92@163.com (Y.C.); weilei5@hotmail.com (L.W.); yiliuxivip@163.com (L.Y.); 2 Institute of Crop Sciences (ICS), Chinese Academy of Agricultural Sciences (CAAS), Beijing 100081, China; bjma055@163.com

**Keywords:** *Beta vulgaris* L., autophagy, genome-wide analysis, low nitrogen, transcriptome

## Abstract

Sugar beet (*Beta vulgaris* L.) is a significant global crop for sugar production, with nitrogen playing a crucial role in its growth, development, and sugar yield. Autophagy facilitates nutrient reabsorption and recycling under nutrient stress by degrading intracellular components, thereby enhancing plant nitrogen use efficiency. However, research on the autophagy response to low-nitrogen stress in sugar beet remains limited. In this study, 29 members of the *ATG* gene family were identified, with genes within the same subfamily displaying similar gene structures and conserved domains. These *ATG* genes in sugar beet contain various hormone and stress-response elements. Transcriptome data and qRT-PCR analysis further revealed that the expression levels of *ATG4*, *ATG8b*, *ATG18a, TOR*, *NBR1*, *ATI, ATG8a*, *ATG12*, and *VTI12a* were significantly upregulated under low-nitrogen stress, with most genes showing high expression levels across different tissues. These *ATG* genes are thus likely involved in regulating autophagy in response to low-nitrogen conditions. The observed increase in autophagosome numbers further supports the induction of autophagy by low-nitrogen stress. These nine genes can be considered key candidates for further research on nitrogen-sensitive autophagy in the sugar beet *ATG* gene family. This study provides a comprehensive analysis of the structure and biological functions of *ATG* genes in sugar beet, offering genetic resources for future efforts to improve sugar beet varieties through genetic engineering. Such efforts could focus on regulating autophagy to enhance nitrogen use efficiency and develop new germplasm.

## 1. Introduction

Nitrogen is an essential element for plant growth and development, serving as a key component of chlorophyll, proteins, and secondary metabolites while also playing a crucial role in determining crop yield [1]. With increasing agricultural demands, nitrogen fertilizer usage is projected to rise, potentially reaching 2.4 million tons by 2050 [2]. However, plants absorb only a small fraction of applied nitrogen, with the majority lost to the environment, resulting in resource wastage and environmental pollution [3]. Thus, improving nitrogen use efficiency has become a critical challenge in agricultural production [4]. A deeper understanding of plant nitrogen metabolism and transport signaling pathways is critical for addressing this issue. Autophagy, a conserved intracellular degradation pathway in eukaryotes, promotes the recycling of macromolecules by degrading them [5]. This process plays a critical role in enhancing nitrogen utilization in plants. Under normal conditions, autophagy functions at a basal level to maintain protein quality control [6]; however, during stress conditions such as nitrogen or carbon starvation, autophagy is greatly induced, increasing plant resilience by promoting the degradation of macromolecules and facilitating nutrient recycling [7].

Autophagy includes three types—microautophagy, macroautophagy, and megaautophagy—each with overlapping functions but distinct components [8]. Macroautophagy is the most extensively studied form, in which autophagosomes enclose cellular contents, transport them to the vacuole, and fuse with the vacuolar membrane to form autophagic bodies. These bodies are then degraded by vacuolar hydrolases [9]. Autophagy can further be categorized into selective and nonselective forms. Selective autophagy degrades specific substrates through receptor recognition, while nonselective autophagy is typically induced by nutritional stress [10]. The autophagic process relies on a series of ATG proteins, and numerous autophagy-related genes have been identified in plants such as *Arabidopsis*, rice, tobacco, poplar, and alfalfa [11,12,13,14,15]. Autophagy can be divided into four functional categories: autophagosome initiation (ATG1 kinase complex); membrane trafficking; autophagosomal membrane modification and vesicle nucleation (PI3K complex); and autophagosome maturation (ATG8/12 ubiquitin-like conjugation system) [16]. These molecular mechanisms collectively regulate the autophagy process, ensuring efficient degradation and recycling of cellular materials.

Autophagy plays a crucial role in responding to nutrient stress by promoting the degradation and recycling of cellular materials, thereby enhancing plant tolerance to low-nitrogen conditions [17,18]. This process is closely associated with nitrogen recycling and the expression levels of *ATG* genes. Studies have shown that in oats, *ATG* genes are upregulated under low-nitrogen conditions, with nitrogen-sensitive materials showing increased autophagic activity and a more pronounced response to nitrogen stress [19]. Similarly, in barley, an increased expression of *ATG* genes has been observed in leaves subjected to low-nitrogen stress [20]. For example, in the *Arabidopsis AtATG5* mutant, premature leaf senescence and reduced nitrogen remobilization and utilization efficiency were observed under both sufficient and low-nitrogen conditions [21]. In maize, the *ZmATG12* mutant displayed impaired autophagy, leading to early leaf senescence and a reduction in the nitrogen harvest index [22]. Conversely, overexpression of *CsATG8e* in tea plants increased autophagic activity, improving nitrogen utilization efficiency [23]. Overexpression of *MdATG18a* in apple plants under low-nitrogen conditions led to increased nitrate content in the leaves and improved plant resistance to low-nitrogen stress [24]. Similarly, research on tomato *SlATG6* has demonstrated that overexpression of autophagy genes can enhance nitrogen assimilation efficiency under low-nitrogen conditions [25]. Collectively, these findings indicate that impaired protein degradation in autophagy mutants results in the failure to recycle cellular contents under nutrient stress, highlighting the critical role of autophagy genes in enhancing plant resistance to nutrient deficiencies.

In summary, *ATG* genes involved in autophagy play diverse roles in plant responses to low-nitrogen stress. However, studies on the role of autophagy genes in sugar beet (*Beta vulgaris* L.) under low-nitrogen stress remain limited. As a biennial crop in the Amaranthaceae family, sugar beet is not only an important global source of sugar but also provides raw materials for industries such as bioethanol production and animal feed, giving it significant economic value [26]. Furthermore, sugar beet growth is highly dependent on nitrogen supply, and sufficient nitrogen is crucial for maximizing sugar yield [27]. While nitrogen fertilizers can enhance sugar production, excessive use increases costs and contributes to environmental issues such as soil compaction and water eutrophication, which negatively affect the sustainable production of sugar beet and soil health. Despite these challenges, the current literature on genome-wide analyses for the identification of *ATG*-related genes in sugar beet remains limited. Most existing studies focus on model plants and species with well-annotated genomes, leading to an incomplete understanding of *ATG* gene functions and regulation in non-model crops such as sugar beet. Additionally, due to potential differences in gene functions across species, cross-species gene function prediction poses difficulties, and the reliability of gene annotation and expression analysis in sugar beet is still under development. Therefore, further research is needed to address these gaps.

In this study, we conducted a genome-wide analysis to identify the *ATG* gene family in sugar beet, examining their physicochemical properties, chromosomal localization, gene structural features, evolutionary relationships, and expression patterns. This research aims to enhance our understanding of autophagy under low-nitrogen conditions, providing a theoretical foundation for future genetic engineering efforts to improve sugar beet varieties.

## 2. Results

### 2.1. Identification of ATG Gene Family in Sugar Beet

**Figure 1:**
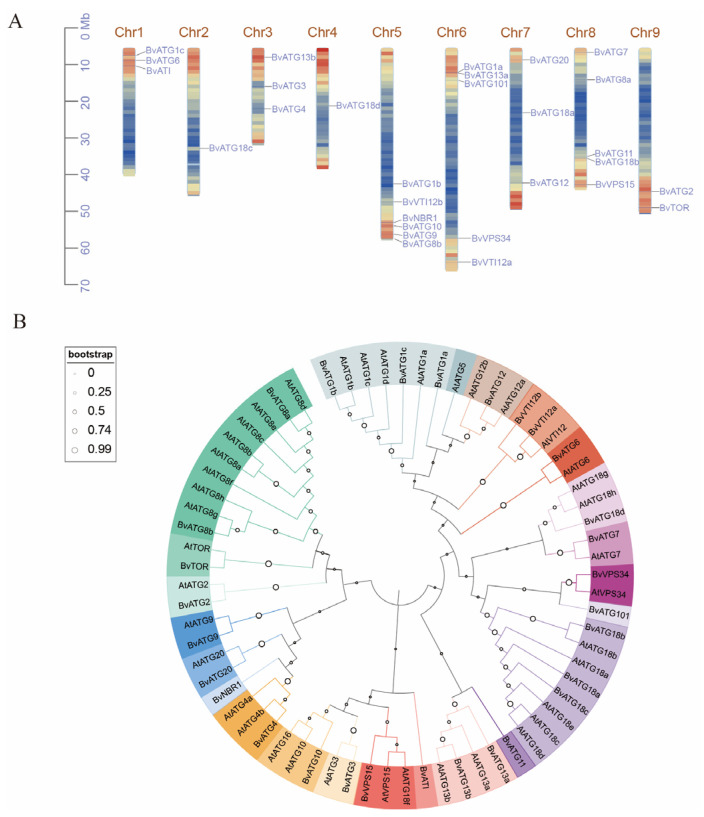
Identification and phylogenetic analysis of *ATG* gene family in sugar beet. (**A**) Chromosomal distribution of *BvATG* family members. (**B**) The phylogenetic tree of the *ATG* gene families from beet and *Arabidopsis* was constructed using the maximum likelihood method in MEGA11, with the bootstrap test set to 1000 iterations.

In this study, a total of 29 autophagy-related genes were identified in sugar beet, named based on their homology with autophagy genes from other species. As shown in Supplementary Table S1, the lengths of the beet *ATG* gene sequences range from 612 to 8025 bp. The molecular weights of the associated proteins span from 10.52 to 277.30 kDa, with isoelectric points ranging from 4.50 to 9.59. Subcellular localization predictions indicated that most of the *ATG* proteins are localized in the nucleus, while a smaller number are found in the chloroplasts, cytoplasm, and vesicles. The *ATG* gene family members in sugar beet are distributed across nine chromosomes., with six genes located on chromosome 5, six genes on chromosome 6, and five genes on chromosome 8. Additionally, three genes are located on chromosomes 1, 3, and 7, two genes on chromosome 9, and one gene each on chromosomes 2 and 4 (Figure 1A).

### 2.2. Phylogenetic and Synteny Analysis of the ATG Gene Family Members in Sugar Beet

**Figure 2:**
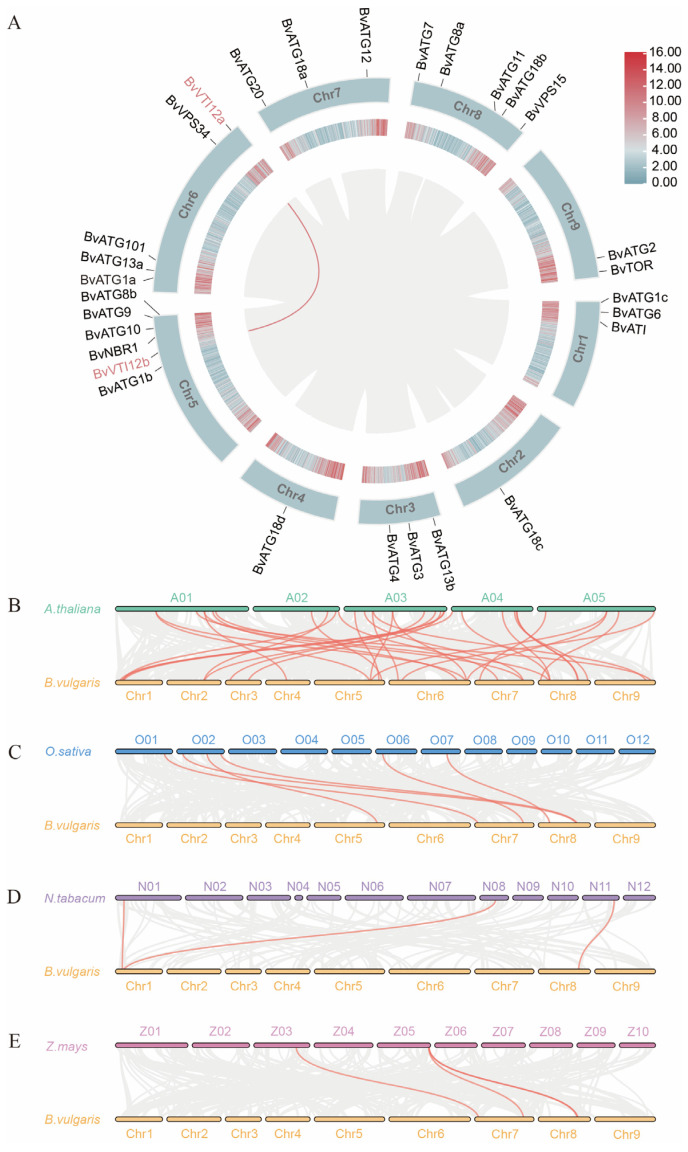
Synteny analysis of the *ATG* gene family in sugar beet. (**A**) *ATGs* gene family intraspecies collinearity analysis. Chromosomes are distinguished by different colors. (**B**) *A. thaliana.* (**C**) *O. sativa.* (**D**) *N. tabacum.* (**E**) *Z. mays.* Different colors are used to represent different species. The gray background represents the synteny between the sugar beet genome and those of other species, while the red lines highlight the synteny of *ATG* gene pairs.

To investigate the evolutionary relationships among *ATG* genes, a phylogenetic tree of *Arabidopsis thaliana* and sugar beet *ATG* genes was constructed using MEGA 11.0 (Figure 1B). The analysis revealed a high degree of similarity between homologous ATG proteins in sugar beet and *Arabidopsis thaliana*, suggesting that orthologous proteins derived from a common ancestor may perform similar functions. As shown in Figure 2A, within-species synteny analysis identified a pair of syntenic genes in the *ATG* family, *BvVTI12a* and *BvVTI12b*, located on chromosomes 6 and 5, respectively. To further elucidate the evolutionary relationships within the *ATG* gene family, synteny analysis was performed, comparing sugar beet with *Arabidopsis thaliana*, rice, maize, and tobacco. The results (Figure 2) revealed 33 pairs of homologous genes between sugar beet and *Arabidopsis thaliana*, 6 pairs with rice, and 3 pairs each with tobacco and maize (Supplementary Figure S1, Table S2). The higher number of homologous gene pairs shared between sugar beet and dicotyledonous species, compared to monocotyledonous species, suggests that certain *BvATG* genes may have been present prior to species divergence. Furthermore, some sugar beet *ATG* genes were found to be absent from syntenic regions with dicotyledonous species, implying that chromosomal rearrangements and fusions during genome evolution may have resulted in selective gene loss in sugar beet.

### 2.3. Gene Structure and Conserved Domain of the ATG Gene Family in Sugar Beet

**Figure 3:**
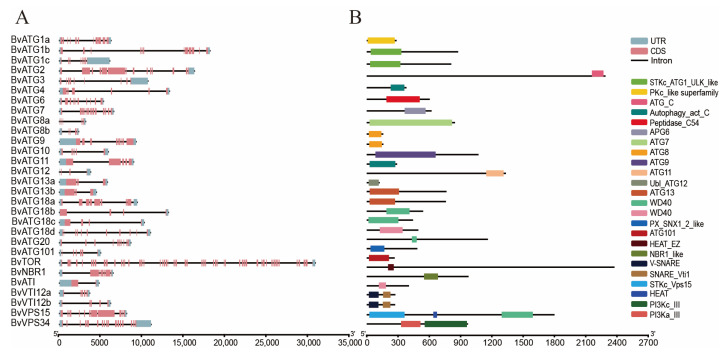
Analysis of the gene structure and conserved domain of the *ATG* family in sugar beet. (**A**) Exons, introns, and untranslated regions (UTRs) of each subgroup are represented by yellow rectangles, black lines, and green rectangles, respectively. The observed differences in exon–intron structures may indicate evolutionary adaptations that contribute to functional diversity within the *ATG* gene family. (**B**) Conserved domains, illustrating the shared structural features that may reflect the conservation of core functions across species.

The differences in intron and exon structures within a gene family play a crucial role in its evolutionary process. As shown in Figure 3A, all *ATG* genes contain introns but the number of exons varies across different subfamilies. This variation highlights the structural diversity among members of the *ATG* gene family in sugar beet. For example, members of the *BvATG8*, *BvATG13*, and *BvVTI12* subfamilies show consistent numbers of exons and introns. Specifically, in the *BvATG1* subfamily, both *BvATG1b* and *BvATG1c* contain 13 exons and 12 introns, while *BvATG1a* has 8 exons and 7 introns. Similarly, *BvATG18b* and *BvATG18c* both possess four exons and four introns, whereas *BvATG18a* and *BvATG18d* differ in their exon and intron counts. These findings suggest that, although CDS sequences within the same subfamily show high homology, evolutionary changes have led to the loss of certain segments in some subfamily members. Among the sugar beet *ATG* gene family, *BvVTI12a*, *BvVTI12b*, and *BvVPS15* each contain two domains, whereas other family members possess only one, emphasizing the variation in conserved domains among different subfamilies. Additionally, *BvATG1*, *BvATG8*, *BvATG13*, *BvATG18*, and *BvVTI12* share the same domain, indicating that domains are typically highly conserved within the same subfamily (Figure 3B).

### 2.4. Identification of Cis-Acting Elements in the Promoter of ATG Gene Family in Sugar Beet

**Figure 4:**
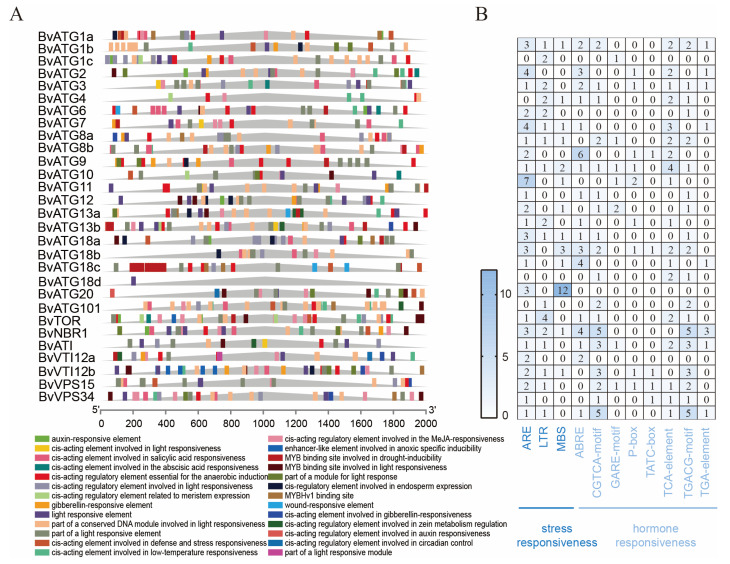
Cis-regulatory elements in the promoter regions of *BvATGs*. (**A**) The number of different types of cis-regulatory elements in each gene. For each *BvATG* gene, the cis-regulatory elements within the 2000 bp region upstream of the predicted ORF were analyzed. (**B**) The number of specific types of cis-regulatory elements related to hormone and stress responses.

To better understand the functional characteristics of the *BvATG* family members, we predicted the cis-acting elements located in the 2000 bp upstream region of the translation start site using the PlantCare database. These elements play a critical role in regulating gene expression. As shown in Figure 4, the *ATG* gene family of sugar beet contains a wide variety of cis-acting elements, including those responsive to auxin, stress, gibberellin, cold, salicylic acid, abscisic acid, jasmonic acid, as well as elements induced by drought. Notably, hormone and stress-responsive elements were the most abundant. These findings suggest that the *ATG* gene family plays an important role in sugar beet’s response to abiotic stresses.

### 2.5. Effects of Low-Nitrogen Stress on Autophagic Structures

**Figure 5:**
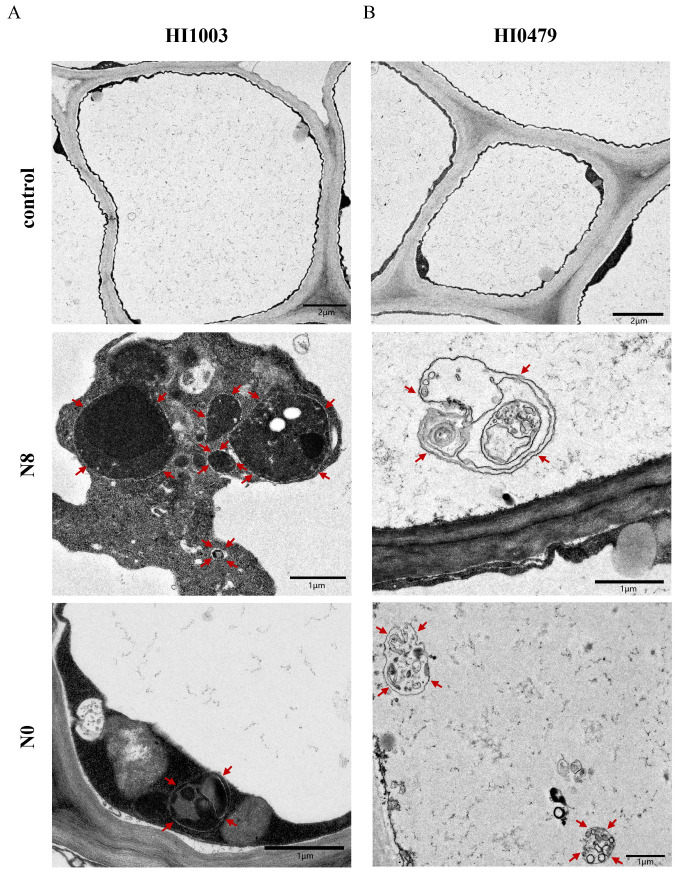
Formation of autophagosomes under low-nitrogen treatment. The autophagic structures are indicated by red arrows. The scale bar is located at the bottom-right corner of each image. (**A**) Autophagic structures of the HI1003 variety under three nitrogen concentrations (N14, N8, N0). (**B**) Autophagic structures of the HI0479 variety under three nitrogen concentrations (N14, N8, N0).

Autophagy is essential for plant responses to nutrient deprivation. To explore the effect of low-nitrogen stress on autophagy, we examined the autophagosome structures in the roots of sugar beet varieties HI1003 and HI0479 during transmission electron microscopy (TEM). This study focused on the accumulation of double-membrane autophagosomes and single-membrane autophagic bodies within the central vacuoles of root cells. The results showed that, following N8 and N0 low-nitrogen treatments, the number of autophagosomes in the roots of HI1003 plants increased compared to the control group (Figure 5A). The roots of HI0479 plants also showed an increase in the number of autophagosomes under low-nitrogen conditions compared to the control (Figure 5B). These findings suggest that plants subjected to low-nitrogen stress produce a higher number of autophagic structures.

### 2.6. GO and KEGG Annotation Analysis of BvATGs

**Figure 6:**
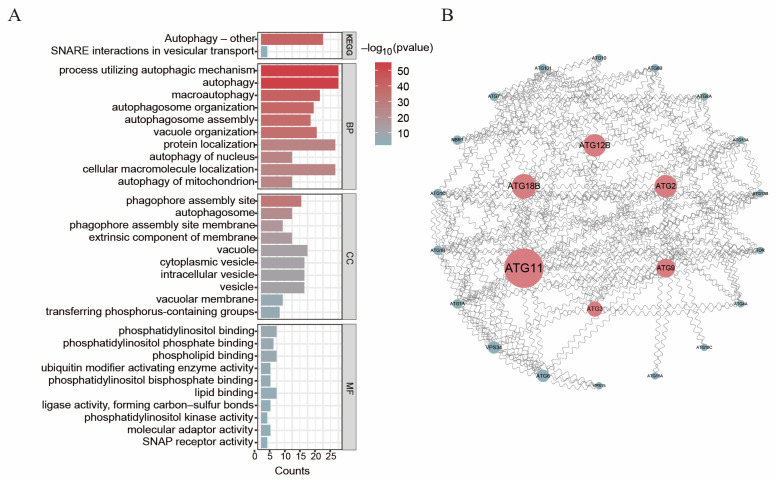
The GO/KEGG enrichment analysis and protein interaction network analysis of the beet ATG gene family. (**A**) GO/KEGG enrichment analysis of the beet *ATG* gene family. (**B**) Protein interaction network analysis.

To investigate the functional roles of *BvATG* genes in sugar beet, we sequenced the transcriptomes of sugar beet root systems subjected to 14 days of low-nitrogen stress and conducted GO annotation for the *BvATGs*. As shown in Figure 6A, the results were classified into three categories: biological processes, cellular components, and molecular functions. In the biological process category, enrichment analysis revealed that *ATG* genes were primarily associated with autophagy-related processes. In the cellular component category, *ATG* genes were predominantly enriched at the phagophore assembly site (GO:0000407) and autophagosome (GO:0005776), with the autophagosome formation process originating at the phagopore assembly site in the cytoplasm. In the molecular function category, *ATG* genes were mainly associated with phosphatidylinositol binding, suggesting their potential involvement in lipid metabolism and signaling pathways related to autophagy (GO:0035091, GO:1901981, and GO:0005543) as well as in ubiquitination modification processes (GO:0008641). Cellular autophagy requires the involvement of ATG-related genes, including the phosphatidylinositol-3 kinase (PI3K) complex and ubiquitination modification system [28]. KEGG enrichment analysis indicated that *BvATGs* were mainly concentrated in the autophagy (ko04136) pathway. These findings suggest that the beet *ATG* genes play a significant role in the autophagy process under low-nitrogen conditions.

### 2.7. Analysis of BvATGs’ Protein Interaction Networks

In Figure 6B, an interaction network of *BvATG* genes was established to analyze their mechanism of action. The results showed that 24 *ATG* genes formed an interaction network with proteins such as *BvATG11*, *BvATG18b*, *BvATG12b*, *BvATG2, BvATG9*, and *BvATG3*, which might play central roles in the interaction process. Based on functional classification in *Arabidopsis*, the network can be divided into four modules. In the first module, *BvATG1*, *BvATG3*, and *BvATG11* interact to form the ATG1 kinase complex. The second module includes two components of the PI3K complex, *BvATG6* and *BvVPS34*. The third module comprises *BvATG2*, *BvATG9*, and *BvATG18b*, which are involved in recruitment membranes for autophagy. The fourth module consists of *BvATG7*, *BvATG8a*, *BvATG8b*, and *BvATG12*, which function as part of the ubiquitin-like conjugation system. The *BvATGs* function similarly to those in *Arabidopsis*, indicating that the beet *ATG* genes may be evolutionarily conserved with the *Arabidopsis ATG* genes.

### 2.8. Analysis of the Expression Pattern of BvATGs

**Figure 7:**
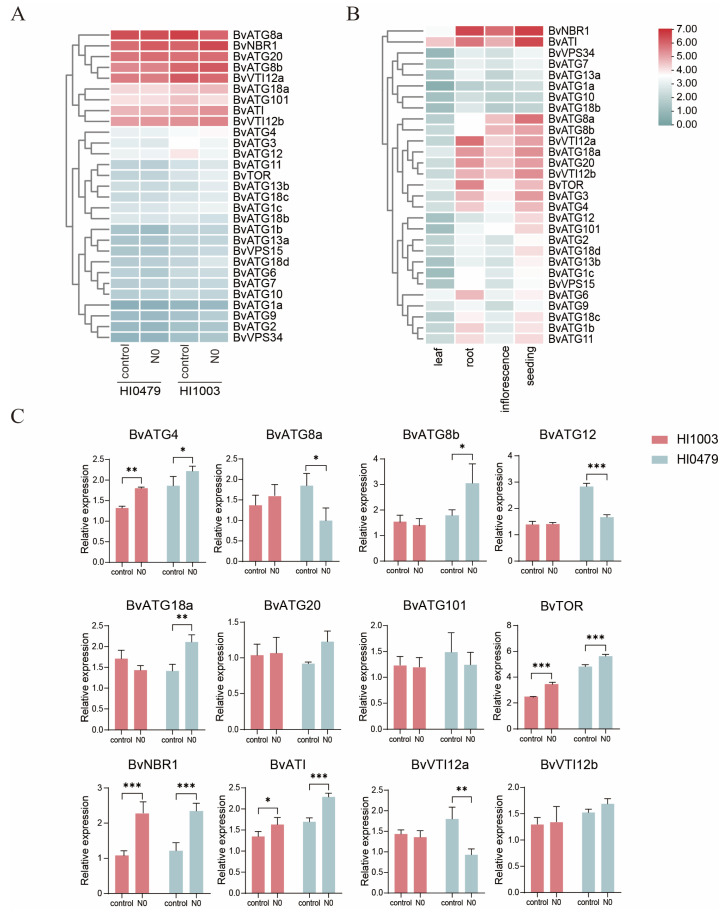
Expression patterns of *BvATGs* under low-nitrogen stress and in different tissues. The color bar represents the normalized values (log_2_(FPKM+1)), ranging from blue (low expression levels) to red (high expression levels). (**A**) Heatmap of *BvATG* transcript abundance under low-nitrogen stress. (**B**) Heatmap of *BvATG* transcript abundance in leaves, roots, flowers, and seeds. (**C**) Relative expression levels of four *BvATG* genes in roots under low-nitrogen stress. Statistical analysis was performed using two-way ANOVA followed by multiple comparisons. “*” indicates a significance level of *p* < 0.05, “**” indicates *p* < 0.01, and “***” indicates *p* < 0.001. The sample size (n) for each bar is *n* = 3.

To explore the expression patterns of *ATG* genes in sugar beet under low-nitrogen stress, transcriptome data from the roots of two sugar beet varieties, HI1003 and HI0479, were analyzed after 14 days of low-nitrogen conditions (Figure 7A). The expression patterns of *BvATG* genes were similar in both varieties under low-nitrogen stress. The expression levels of 12 *ATG* genes increased in both varieties, with 19 and 20 *ATG* genes showing upregulated expression in HI0479 and HI1003, respectively, while other *ATG* genes showed no significant changes. The results indicate that under low-nitrogen stress, most *ATG* genes were upregulated to varying degrees, though some gene family members exhibited relatively weak responses. Notably, two members of *BvATG8* and *BvVTI12*, as well as members of *BvNBR1*, *BvATG20*, *BvATG18a*, *BvATG101*, and *BvATI* showed higher expression levels, suggesting that these *ATG* genes may play crucial regulatory roles in the autophagy process under low-nitrogen conditions. Additionally, we analyzed the expression patterns of *ATG* genes across various sugar beet tissues (Figure 7B). Most *ATG* genes exhibited low expression levels in leaves but were generally expressed in seeds, roots, and flowers. Two members of *BvATG8* and two members of *BvVTI12*, *BvATG18a*, *BvATG20*, *BvTOR*, *BvATG3*, and *BvATG4* showed similar expression patterns in seeds, roots, and flowers, with higher expression levels compared to leaves, while *BvNBR1* and *BvATI* exhibited consistently higher expression across different tissues. These *ATG* genes may play essential roles in the autophagy process and contribute to the growth and development of sugar beet.

### 2.9. qRT-PCR Validation of BvATG Gene Involvement in Low-Nitrogen Stress Response

Building on these predictions, 12 *ATG* genes were selected for further analysis, and their expression levels in roots under low-nitrogen stress were examined using qRT-PCR (Figure 7C). The results showed that under low-nitrogen conditions, the expression levels of *BvATG4*, *BvNBR1*, *BvTOR* and *BvATI* were significantly elevated in both varieties. In HI0479, *BvATG8b* and *BvATG18a* exhibited significant upregulation, while *BvATG8a*, *BvATG12*, and *BvVTI12a* were significantly downregulated. These findings suggest that these nine *ATG* genes play key regulatory roles in the autophagy process during sugar beet’s response to low-nitrogen stress, with functional differences among them.

## 3. Discussion

Autophagy, a highly conserved intracellular degradation pathway in eukaryotes, is a protective mechanism that enables cells to respond to nutrient deficiencies. It plays an important role in degrading damaged proteins and promoting the recycling and reuse of cytoplasmic components, thereby helping to maintain intracellular homeostasis and function [29]. In this study, we identified 29 autophagy-related genes within the sugar beet genome. The number of *ATG* genes in sugar beet is comparable to the 33 found in rice and 30 in tobacco, suggesting that autophagy genes are highly conserved across evolutionary lineages. However, the number of *ATG* genes in sugar beet is fewer than the 47 in *Arabidopsis*, 48 in poplar, and 39 in alfalfa. This difference may be attributed to variations in subfamily copy numbers across species. For example, sugar beet has 2 copies of the *ATG8* gene, while *Arabidopsis*, poplar, and alfalfa have 9, 11, and 8 members, respectively. Similarly, sugar beet has 4 copies of the *ATG18* gene, compared to 8, 10, and 8 members in *Arabidopsis*, poplar, and alfalfa, respectively. This indicates that the *ATG* subfamily in sugar beet is contracted, with no significantly amplified subfamilies.

Comparative phylogenetic analysis with the *Arabidopsis ATG* gene family revealed a high degree of similarity between the *ATG* genes of sugar beet and those in different subfamilies of *Arabidopsis*. Additionally, protein interaction network analysis indicated that the *ATG* genes in sugar beet and *Arabidopsis* exhibit similar interaction patterns. These results suggest that the autophagy process is highly conserved across species and that *ATG* genes likely perform similar functions in different species, a conclusion consistent with previous findings on *ATG* genes in Chinese Cabbage [30]. Based on the collinearity analysis, all *ATG* genes within sugar beet and across other species demonstrate collinearity, reflecting the close evolutionary relationships of *ATG* genes among species. In both poplar and Chinese Cabbage, members of the *ATG* subfamily typically exhibit consistent numbers of exons and introns, including *ATG8*, *ATG13*, and *VTI12*, among others. Notably, *TOR* has the highest count of exons and introns, a trend also observed in our study [14,30]. However, the number of exons and introns in certain members of *ATG1* and *ATG18* subfamilies in sugar beet shows some variation. For example, beet *ATG1a* has 8 exons, while *ATG1* in both poplar and Chinese Cabbage contains 13 exons, a difference that may be attributed to the insertion and removal of introns during the evolution of sugar beet *ATG* genes. The functional significance of this structural variability within the *ATG* gene subfamilies is particularly important. Differences in exon–intron structures can impact gene transcription regulation, alternative splicing, and protein expression, leading to varied physiological functions and abilities to respond to environmental stress. Under nitrogen stress conditions, such structural diversity may provide plants with different adaptive strategies to modulate autophagy responses and optimize nitrogen uptake and utilization. Different subfamilies of sugar beet *ATG* genes have similar conserved structural domains, such as *ATG8*, *ATG13*, *ATG18*, and *VTI12*, which is consistent with findings in buckwheat [31]. These subfamilies may exhibit functional redundancy, and there may also be specificity in their expression patterns, a hypothesis that was later confirmed through transcriptional profiling of expression levels.

The autophagy *ATG* gene family plays a crucial role in plant growth, development, and stress responses. In sugar beet, numerous hormone-responsive and stress-responsive elements have been identified within the *ATG* gene family. Similarly, stress-related response elements have also been detected in the promoter regions of *Arabidopsis ATG* genes. For example, the *AtATG9* and *RNAiATG18* mutants in *Arabidopsis* showed early senescence under both sufficient- and low-nitrogen conditions. Additionally, the *AtATG14a/aATG14b* double mutant exhibits nitrogen stress intolerance, premature senescence, and reduced material recycling efficiency [32]. Different expressions of *ATG* genes under stress conditions have also been observed in other plants. In cereals, *ATG* genes displayed distinct expression patterns following hormone treatments, with two members of *ATG7* being rapidly upregulated. Moreover, a large number of *ATG* genes were found to be upregulated in response to salt, drought, and cold stress, with their expression levels of *ATG* genes increasing significantly in the later stages of drought stress. During the early stage of cold stress, *ATG* gene expression levels decreased, with only seven *ATG* genes showing increased expression after 24 h [33]. In tea plants, 10 of the 12 *ATG* genes were upregulated after low-nitrogen stress, although *ATG8b* was downregulated [23]. Overall, these findings suggest that *ATG* genes in sugar beet are essential for responding to low-nitrogen stress.

GO and KEGG enrichment analyses confirmed that *ATG* genes are mainly involved in the autophagy process. Under low-nitrogen stress, the expression patterns of *ATG* genes were similar between the two sugar beet varieties, with 9 genes showing higher expression and 20 genes showing lower expression. However, most *ATG* genes demonstrated some degree of increased expression. Similarly, in tomato, the expression of *ATG* genes increases during the early stages of low-nitrogen stress, then declines after 12 days, suggesting that *ATG* genes play a role in the nitrogen stress response and contribute to nitrogen tolerance [25]. qRT-PCR quantification of 12 highly expressed *ATG* genes revealed that 6 genes (*BvATG4*, *BvATG8b*, *BvATG18a*, *BvTOR*, *BvNBR1*, and *BvATI*) were significantly upregulated in the roots of sugar beet under low-nitrogen stress, while 3 genes (*BvATG8a*, *BvATG12*, and *BvVTI12a*) were significantly downregulated. These findings imply that these nine genes may play key roles in sugar beet’s response to low-nitrogen stress. Most of these genes, except *BvATG12*, were highly expressed in roots, flowers, and seeds, with *BvNBR1* and *BvATI* showing the highest expression across different tissues. *BvATG4*, *BvATG8*, and *BvATG12* are part of the ubiquitin-like system that serves as a key switch for activating autophagy [34]. *NBR1*, a ubiquitinated protein aggregate receptor, mediates autophagy by binding to ATG8 on the autophagosome membrane [35]. *ATG18* is crucial for regulating autophagy and vacuole morphology [36], while *ATI,* a plant-specific protein, interacts with *ATG8* and is degraded in vacuoles under nutrient stress, accelerating nutrient recycling [37]. *TOR*, a negative regulator of the ATG1 kinase complex, inhibits autophagy under nutrient-rich conditions but promotes autophagosome expansion and closure under nutrient stress, thereby activating autophagy [38]. *VTI12*, a plant-specific SNARE protein, is essential for autophagosome–vacuole membrane fusion, and *Arabidopsis* T-DNA insertion mutants of *VTI12* display accelerated senescence and impaired autophagy under nutrient-deprived conditions [39]. Autophagy is crucial for plants’ adaptation to nutrient starvation by inducing autophagosome formation, which sequesters denatured proteins and transports them to vacuoles for degradation, facilitating nutrient recycling [7]. The significant expression changes observed in these nine *ATG* genes under low-nitrogen stress suggest that they regulate the autophagy process in response to nitrogen deficiency in sugar beet. Additionally, the increase in autophagosome numbers observed in both varieties under low-nitrogen stress indicates that autophagy is induced, resulting in heightened autophagic activity in sugar beet. Given the crucial role of the *ATG* gene family in sugar beet’s response to low-nitrogen stress, further in-depth studies focusing on the functional roles and interaction mechanisms of key *ATG* genes—such as *BvATG4*, *BvATG8b*, *BvATG18a*, *BvTOR*, *BvNBR1*, *BvATI*, *BvATG8a*, *BvATG12*, and *BvVTI12a*—will provide valuable insights into the regulatory networks involved. Such research will enhance our understanding of how autophagy affects nitrogen use efficiency in crops, offering a theoretical basis for improving nitrogen utilization through autophagic regulation.

Currently, the world is facing challenges such as nitrogen deficiency, excessive application of nitrogen fertilizers, and low-nitrogen use efficiency, which severely impact the yield and quality of crops. Autophagy plays a critical role under nutrient-deficient conditions by degrading and recycling macromolecules, thus promoting nutrient absorption and reutilization. This study revealed the crucial role of autophagy-related genes in sugar beet under nitrogen stress, providing a theoretical basis for crop genetic improvement. Applying these genes in breeding programs could enhance crop productivity and nutrient use efficiency in low-nitrogen environments. However, there are obstacles to the practical application of these findings. Different sugar beet varieties exhibit varied regulatory responses to autophagy genes, which may result in inconsistencies across genotypes. Additionally, the reproducibility of greenhouse results under actual field conditions may be influenced by environmental factors such as soil type and climate variability.

Future research should focus on integrating these genes under field conditions to validate their effectiveness and stability. Furthermore, the application of these genes in molecular breeding and gene editing technologies to select and develop low-nitrogen-tolerant sugar beet varieties holds significant agricultural and environmental implications. Moreover, the transport and utilization of nitrogen within plants are regulated by systemic signals, such as cytokinins and peptides. Therefore, whether there is a more direct relationship between autophagy-mediated nitrogen absorption and assimilation and these hormones and signaling molecules warrants further investigation.

## 4. Materials and Methods

### 4.1. Plant Materials and Treatment

The sugar beet high-yield variety HI1003 and high-sugar variety HI0479 used in this study were maintained by the College of Agriculture at Inner Mongolia Agricultural University (Hohhot, China) and were sown on 30 August 2023. Plump seeds were selected and planted in seedling pots filled with a mixture of vermiculite, soil, and sand in a 1:1:1 ratio. The pots were placed in an artificial climate chamber with a 16/8 h (day/night) photoperiod and an average day/night temperature of 25/20 °C. After seedling emergence, Hoagland nutrient solution was applied once to each pot, and low-nitrogen treatment was initiated when the first pair of true leaves had fully expanded. The experiment included three nitrogen concentration treatments: N0 (0 mmol/L), N8 (8 mmol/L), and a control group N14 (14 mmol/L). Hoagland nutrient solution at different concentrations was applied every 7 days. After 14, 21, and 28 days of low-nitrogen treatment, the underground parts of the plants were collected, rapidly frozen in liquid nitrogen, and stored at −80 °C for further analysis.

The low-nitrogen treatment and the nutrient solution applied to control N14 was modified Hoagland nutrient solution [40]. It contained Ca(NO_3_)_2_ 4 mmol·L^−1^, KNO_3_ 6 mmol·L^−1^, CaCl_2_ 4 mmol·L^−1^, KH_2_PO_4_ 2 mmol·L^−1^, and MgSO_4_ 1.5 mmol·L^−1^. N8 was treated with Ca(NO_3_)_2_ 2 mmol·L^−1^, KNO_3_ 4 mmol·L^−1^ as nitrogen source, and N0 was treated with no nitrogen source. Calcium and potassium were replaced with CaCl_2_, KH_2_PO_4_, and KCl. The pH was adjusted to about 6.0 with NaOH.

### 4.2. Identification and Phylogenetic Analysis of ATG Gene Family in Sugar Beet

The genome and genome annotation files for *Arabidopsis* thaliana and sugar beet were downloaded from the Ensembl website (https://plants.ensembl.org/index.html, accessed on 26 July 2024). A local BLAST database was created and the protein sequences of autophagy-related *ATG* genes from previously reported studies in *Oryza sativa* (rice), *Arabidopsis thaliana*, *Nicotiana tabacum* (tobacco), and *Zea mays* (maize) were retrieved to serve as query sequences. BLASTP searches were conducted with an E-value cutoff of less than 1 × 10^−5^, ensuring sequence similarity percentages greater than 70%. To increase the robustness of the results, we set a minimum sequence coverage of 80% for target genes. All identified homologous sequences were uploaded to the InterPro database for domain analysis to confirm the presence of ATG domains. Additionally, the SMART database (http://smart.embl-heidelberg.de/, accessed on 28 July 2024) was used to confirm the presence of ATG conserved domains in the filtered protein sequences.

The ATG protein sequences from *Arabidopsis* and sugar beet were aligned using the MUSCLE algorithm in the MEGA11 software. A phylogenetic tree was constructed using the maximum likelihood method with 1000 bootstrap replicates. The phylogenetic tree was beautified using iTOL (https://itol.embl.de/, accessed on 28 August 2024). The molecular weight (Mw) and isoelectric point (pI) of the proteins encoded by the ATG genes in sugar beet were estimated using the ProtParam tool on the Expasy website(https://web.expasy.org/protparam/, accessed on 31 July 2024), and the subcellular localization of the ATG gene family was evaluated using Cell-PLoc 2.0 (http://www.csbio.sjtu.edu.cn/bioinf/plant/, accessed on 31 July 2024) [41].

### 4.3. Characteristic Analysis of the ATG Gene Family in Sugar Beet

Cis-acting element analysis was performed on the 2000 bp sequence upstream of the gene start codon using PlantCare (https://bioinformatics.psb.ugent.be/webtools/plantcare/html/, accessed on 10 August 2024) [42]. The cis-acting elements of *ATG* gene family members were visualized using TBtools software(TBtools–II v2.138). Fragmentary and tandemly duplicated genes within the *ATG* gene family between sugar beet, *Arabidopsis* thaliana, rice, and tobacco were analyzed using MCScanX, and the synteny relationships of the *ATG* gene family among these species were visualized using TBtools.

### 4.4. Analysis of BvATGs’ Gene Structure, Chromosomal Mapping, and Conserved Domains

Based on the position, distribution, and chromosome size of *ATG* genes in sugar beet, chromosomal mapping and visualization of *ATG* gene family members were carried out using TBtools. The CDS sequences, along with genomic sequences including introns and exons of the *ATG* gene family were analyzed and visualized using TBtools. Conserved domains of the *ATG* genes were predicted using the NCBI-CDD (https://www.ncbi.nlm.nih.gov/Structure/cdd/wrpsb.cgi, accessed on 25 August 2024), and visualized using TBtools.

### 4.5. Transmission Electron Microscope Analysis

After 14 days of low-nitrogen treatment, root samples from the control group (N14) and the N8 and N0 treatments were cut into approximately 1 mm × 1 mm pieces. The samples were vacuum-fixed in 2.5% glutaraldehyde at 4 °C for 4 h, followed by fixation with 1% osmium tetroxide, and then rinsed with 0.1 mol/L phosphate buffer. The samples were subsequently dehydrated using a graded ethanol series, embedded in epoxy propane and embedding solution, and polymerized at 60 °C for 48 h. Ultrathin sections were prepared using an ultramicrotome (Leica UC7, Germany), double-stained with uranyl acetate and lead citrate. The stained sections were placed in a transmission electron microscope (Hitachi HT7800, Tokyo, Japan), observed at an accelerating voltage of 80–120 kV with a magnification of 2500× to 10,000×, and photographed [43].

### 4.6. RNA-Seq Analysis

After 14d of low-nitrogen (N0) treatment, the roots of sugar beet HI1003 and HI0479 were collected, snap-frozen in liquid nitrogen, and stored at −80 °C. Total RNA was extracted from sugar beet roots using an RNA kit (Tiangen, Beijing, China). Following RNA extraction, purification, and library construction, the libraries were subjected to paired-end (PE) sequencing using Next-Generation Sequencing (NGS) on the Illumina platform. The sequencing data were quality controlled using fastp (version 0.23.0) [@fastp]. This process involved removing splice sequences from the reads, filtering out reads shorter than 15 bp, discarding low-quality reads (where the proportion of bases with a quality score below Q20 was greater than 8%), and removing reads containing “N” in the sequence. Gene expression levels were calculated using the FPKM method, and differentially expressed genes were identified based on the criteria of absolute logFC > 1 and *p*-value < 0.05. The transcriptome sequencing was conducted by Wuhan Meitville Biotechnology Co. (Wuhan, China).

GO enrichment analysis and KEGG enrichment analysis of sugar beet ATG genes were performed using Gene Ontology (https://www.geneontology.org/, accessed on 23 August 2024) and KEGG (https://www.genome.jp/kegg/, accessed on 23 August 2024). Protein interaction networks of differentially expressed genes were analyzed using the STRING protein interactions database (https://cn.string-db.org/, accessed on 25 August 2024) and visualized with Cytoscape v 3.7. Transcriptomic data were analyzed to obtain FPKM values of BvATGs in HI1003, HI0479, and across different tissues, with heat maps generated using TBtools. For further details on downloading transcriptome data from various sugar beet tissues, refer to Supplementary Table S3.

### 4.7. Quantitative Real-Time PCR (qRT-PCR)

Total RNA from the lower part of the beet field was extracted using an RNA extraction kit (Tiangen, China) and reverse-transcribed into cDNA using SynScript^®^III RT SuperMix for qPCR (tsingke, Beijing, China). The cDNA was then subjected to qRT-PCR using ArtiCanCEO SYBR qPCR Mix (tsingke, China). Actin was used as the internal reference gene. The reaction system had a total volume of 20 μL, which included 10 μL of PCR mix, 1.6 μL of upstream and downstream primers, 1 μL of cDNA, and 7.4 μL of ddH2O. Gene expression data were calculated using the 2^−ΔΔCt^ method, with each reaction repeated three times. The primers used for qRT-PCR analysis are listed in Supplementary Table S4.

## 5. Conclusions

In this study, 29 *ATG* genes were identified in sugar beet, distributed across 9 chromosomes, with subfamily members exhibiting similar gene structures and conserved domains. Further analysis revealed a substantial number of hormone and stress-response elements within these sugar beet *ATG* genes, suggesting their potential roles in responding to abiotic stress. GO and KEGG enrichment analyses indicated that the *ATG* genes in sugar beet play critical roles in the autophagy process. Expression pattern and level analyses demonstrated that *ATG4*, *ATG8b*, *ATG18a*, *TOR*, *NBR1*, *ATI*, *ATG8a*, *ATG12*, and *VTI12a* showed significant changes in expression under low-nitrogen stress, with most genes highly expressed across various tissues. Additionally, the number of autophagosomes in sugar beet cells increased under low-nitrogen stress. These findings suggest that autophagy activity in sugar beet is enhanced during low-nitrogen conditions and that these *ATG* genes may play key regulatory roles in this process. This study provides a theoretical foundation for improving nitrogen use efficiency in crops through the regulation of autophagy via genetic engineering. To further promote the practical application of these research findings, future studies should focus on validating the functional stability and effectiveness of these genes under field conditions and exploring how these genes can be applied to the genetic improvement of sugar beet and other crops to enhance their tolerance to low-nitrogen conditions and improve nitrogen use efficiency.

## Data Availability

The data that support the finding of this study are available from the corresponding author upon reasonable request.

## Supplementary Materials

The following supporting information can be downloaded at: https://www.mdpi.com/article/10.3390/ijms252211932/s1.
