# Supplementary material for: Genome-Wide and Transcriptome Analysis of Autophagy-Related ATG Gene Family and Their Response to Low-Nitrogen Stress in Sugar Beet
Source: Int J Mol Sci. 2024 Nov 6;25(22):11932. doi: 10.3390/ijms252211932 (PMC11594104; doi:10.3390/ijms252211932)
Supplement: Supplementary file 1 [file ijms-25-11932-s001.zip › Table S1.pdf]

Table S1. Property analysis of the BvATGs proteins in sugar beet.

Gene name	Gene ID	Mw (kDa)	pI	Length of gene(bp)	Predicted location
BvATG1a	BVRB_6g133110	26.62	5.82	1475	Nucleus
BvATG1b	BVRB_5g112680	81.09	6.31	2368	Nucleus
BvATG1c	BVRB_1g001970	75.10	5.63	2637	Nucleus
BvATG2	BVRB_9g218760	213.15	5.36	6176	Chloroplast. Nucleus
BvATG3	BVRB_3g056580	35.39	4.71	1383	Nucleus
BvATG4	BVRB_3g059550	54.48	5.44	2139	Chloroplast. Nucleus
BvATG6	BVRB_1g003180	58.37	5.67	1977	Nucleus
BvATG7	BVRB_8g181770	78.35	5.31	2534	Nucleus
BvATG8a	BVRB_8g186120	14.13	8.61	815	Cytoplasm. Nucleus
BvATG8b	BVRB_5g122250	14.32	5.42	710	Cytoplasm
BvATG9	BVRB_5g120960	103.21	6.27	3518	Cell membrane. Chloroplast. Cytoplasm
BvATG10	BVRB_5g118950	27.20	5.12	1313	Cell membrane. Chloroplast. Nucleus
BvATG11	BVRB_8g191690	126.10	5.59	4095	Nucleus
BvATG12	BVRB_7g170070	10.52	8.89	612	Cytoplasm
BvATG13	BVRB_3g050480	70.77	9.24	3024	Nucleus
BvATG13	BVRB_6g133120	70.62	8.91	2817	Nucleus
BvATG18a	BVRB_7g164090	49.26	7.61	1711	Nucleus
BvATG18b	BVRB_8g191900	40.07	6.55	1903	Nucleus
BvATG18c	BVRB_2g038550	45.29	8.27	1849	Nucleus
BvATG18d	BVRB_4g083240	105.84	5.61	3882	Nucleus
BvATG20	BVRB_7g159050	46.08	6.79	1737	Nucleus
BvATG101	BVRB_6g134680	25.32	6.32	1194	Chloroplast
BvTOR	BVRB_9g222650	277.30	6.59	8025	Chloroplast
BvNBR1	BVRB_5g118120	90.13	5.05	3073	Nucleus
BvATI	BVRB_1g004460	36.91	4.50	1627	Nucleus
BvVTI12a	BVRB_6g153340	25.34	8.77	1045	Vacuole
BvVTI12b	BVRB_5g114280	24.91	9.59	1224	Vacuole
BvVPS15	BVRB_8g197640	170.95	6.10	5168	Nucleus
BvVPS34	BVRB_6g148700	93.19	6.64	3237	Nucleus
