# Supplementary material for: Genome-Wide and Transcriptome Analysis of Autophagy-Related ATG Gene Family and Their Response to Low-Nitrogen Stress in Sugar Beet
Source: Int J Mol Sci. 2024 Nov 6;25(22):11932. doi: 10.3390/ijms252211932 (PMC11594104; doi:10.3390/ijms252211932)
Supplement: Supplementary file 1 [file ijms-25-11932-s001.zip › Table S2.pdf]

Table S2 Synteny analysis of the ATG gene family in different species

**Number**	**Gene name**
a	BvNBR1
b	BvATI
c	BvATG18d
c	BvVTI12a
c	BvVTI12b
c	BvVPS34
c	BvATG9
c	BvATG18a
c	BvATG1c
c	BvATG13a
c	BvATG101
c	BvATG2
c	BvTOR
c	BvATG7
c	BvVPS15
c	BvATG6
c	BvATG4
c	BvATG18c
c	BvATG10
c	BvATG13b
d	BvATG8a
e	BvATG12
e	BvATG20
e	BvATG11
f	BvATG18b
