# Supplementary material for: Genome-Wide and Transcriptome Analysis of Autophagy-Related ATG Gene Family and Their Response to Low-Nitrogen Stress in Sugar Beet
Source: Int J Mol Sci. 2024 Nov 6;25(22):11932. doi: 10.3390/ijms252211932 (PMC11594104; doi:10.3390/ijms252211932)
Supplement: Supplementary file 1 [file ijms-25-11932-s001.zip › Table S3.pdf]

Table S3 Transcriptome data

Tissues	Numbering
SRX287608	leaf
SRX287610	root
SRX287611	inflorescence
SRX287614	seed
