# Supplementary material for: Genome-Wide and Transcriptome Analysis of Autophagy-Related ATG Gene Family and Their Response to Low-Nitrogen Stress in Sugar Beet
Source: Int J Mol Sci. 2024 Nov 6;25(22):11932. doi: 10.3390/ijms252211932 (PMC11594104; doi:10.3390/ijms252211932)
Supplement: Supplementary file 1 [file ijms-25-11932-s001.zip › Table S4.pdf]

Table S4 Primer sequences

Gene name	Forward primers (F)	Reverse primers (R)
BvActin	CGTGGATCTCTTTGGAAGACC	TCTTAAACCACTTCTCACCTTCTCA
BvATG4	ATTCCACAGCAATGTGGTCC	GCGGAGCACCATTTGATTCAT
BvATG8a	ATCCTGATCGAGTCCCTGTGA	CCGGAGCTAGTCGAAGTCTG
BvATG8b	ACCTTTTCGTGTTCTGGAGAGA	TAATGCGAGCTGCTTCTGCT
BvATG12	GCTTCCGAATCACCAAGTGC	TTACCATCAATTCCAAAGTTCTTGA
BvATG18a	TGATTCGAGGATTGCCTGCT	CTGCACCTCTTCTTACCTCCTG
BvATG20	AGGCAAGGAGAAGCCTATCG	ATTGTCCCAGCTCTCTGTGC
BvATG101	GTGTCACTTCGAGAGGTCCTG	ACGTAGTATCCGATGAGCTTGG
BvTOR	TATGTTGGGTGGTGCCCATT	GTATCATGCGCCCTCATTGC
BvNBR1	GCAGCATCTGCTTTGAACGA	GGACGCGTACAATGGGATCAT
BvATI	CTCACCGATTTTTCTATGGCTGA	GCCATCTTGGGAGCTTCTTG
BvVTI12a	GGAGACAGAAGAGTTGGGTGTT	TGTCTACCTCATGCAGCTTCG
BvVTI12b	AGATGCACTGATGGCTTCTG	ATTCTGCTCTGCCTAACCCT
