# Supplementary figures and images for: Genome-Wide and Transcriptome Analysis of Autophagy-Related ATG Gene Family and Their Response to Low-Nitrogen Stress in Sugar Beet
Source: Int J Mol Sci. 2024 Nov 6;25(22):11932. doi: 10.3390/ijms252211932 (PMC11594104; doi:10.3390/ijms252211932)

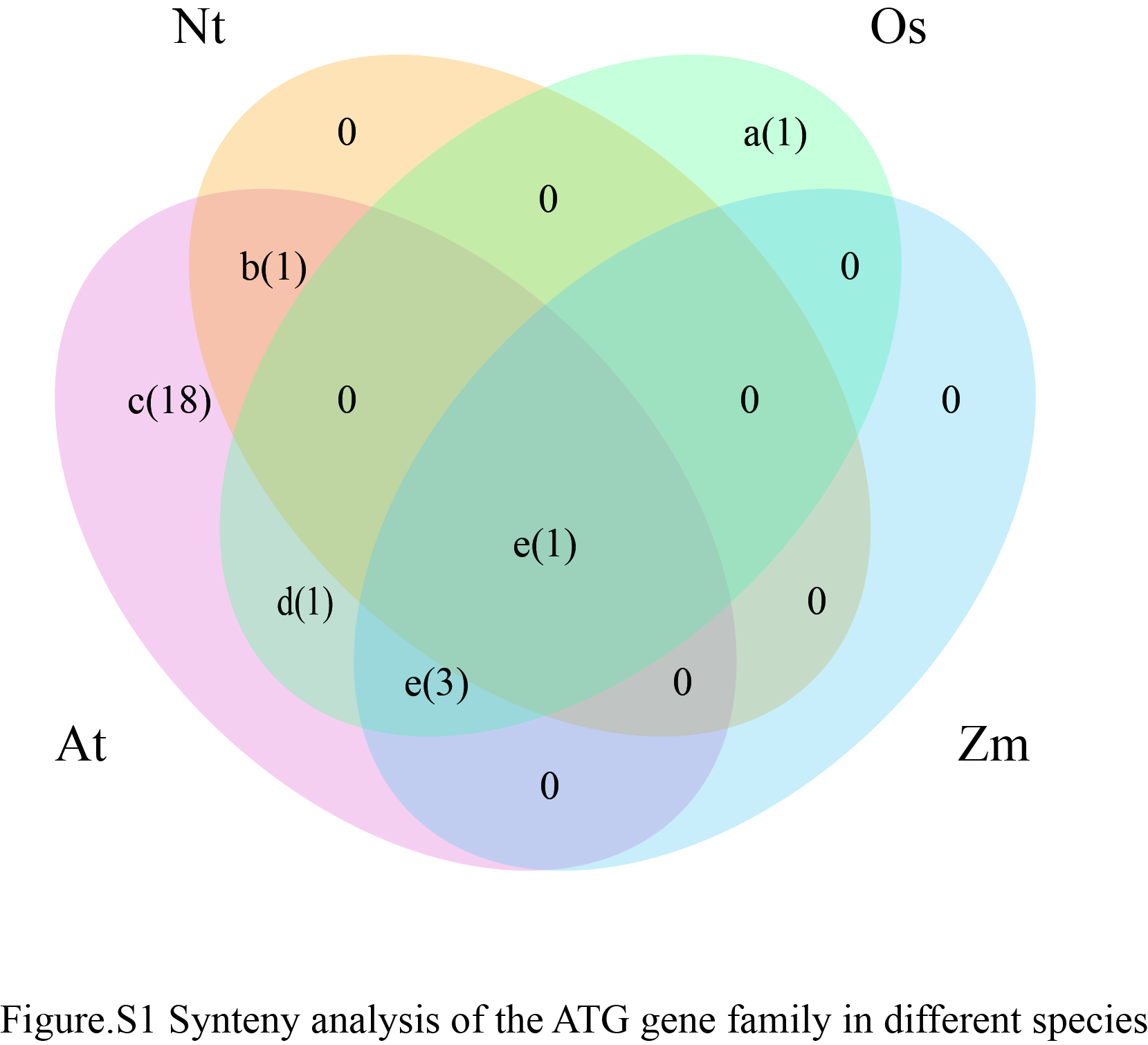

Supplement: Supplementary file 1 [file ijms-25-11932-s001.zip › Figure.S1.jpg]
